# Recurrence Rates and Its Associated Factors after Early Spinal Instrumentation for Pyogenic Spondylodiscitis: A Nationwide Cohort Study of 2148 Patients

**DOI:** 10.3390/jcm11123356

**Published:** 2022-06-11

**Authors:** Jeong Seop Lim, Tae-Hwan Kim

**Affiliations:** Spine Center, Department of Orthopedics, Hallym University Sacred Heart Hospital, Hallym University College of Medicine, Anyang 14068, Korea; yellowberry@hallym.or.kr

**Keywords:** pyogenic spondylodiscitis, vertebral osteomyelitis, early instrumentation, early surgery, recurrence, multiple surgeries

## Abstract

Recent studies have consistently reported the safety and effectiveness of early spinal instrumentation for pyogenic spondylodiscitis. However, none of these studies investigated the recurrence rate or associated factors based on this specific group of patients. Recurrence prediction models that are not based on a homogenous cohort of patients undergoing early spinal instrumentation show theoretical limitations for clinical use. A nationwide, population-based, retrospective cohort study using a claims database was planned to investigate the recurrence rate and its associated factors in patients who underwent early instrumented spinal fusion surgery for pyogenic spondylodiscitis. We used data from the Korean National Health Insurance claims database collected between 2014 and 2018. A total of 2148 patients who underwent early (within 6 weeks after the diagnosis) instrumented spinal fusion surgery for pyogenic spondylodiscitis were included, including 1925 patients (90%) without recurrence and 223 patients (10%) with recurrence. Logistic regression models were used to identify factors associated with recurrence, and sensitivity analysis was performed according to two different definitions for recurrence: shorter (2 weeks or more) and longer (6 weeks or more) periods of antibiotic therapy for recurrence. The recurrence rates in our cohort were 14.1%, 10.4%, and 8.0% at 2, 4, and 6 weeks, respectively, in relation to the duration of antibiotic treatment for recurrence. We identified age 60–69 years (OR = 2.42), age > 70 years (OR = 2.29), posterior thoracic approach (OR = 1.85), multiple surgical approaches (OR = 4.17), use of a cage (OR = 1.66), transfusion (OR = 2.55), antibiotics for resistant organisms (OR = 2.55), and systemic steroid treatment over 2 weeks (OR = 2.27) as the factors associated with recurrence. The recurrence rates and associated factors from our first population-based and the largest clinical study can be practically used as a reasonable reference and provide clinicians with an acceptable tool for the comprehensive risk assessment for recurrence after early spinal instrumentation for pyogenic spondylodiscitis.

## 1. Introduction

Recent studies on pyogenic spondylodiscitis have consistently reported the safety and effectiveness of early spinal instrumentation in comparison with other treatment methods, including conservative treatment or non-instrumented surgery [1,2,3,4,5]. However, despite the similar recurrence rates in patients undergoing early spinal instrumentation and those undergoing other treatment methods, recurrence after early spinal instrumentation is associated with detrimental clinical outcomes and a much higher risk of subsequent recurrence and repetitive spinal surgeries, including surgeries for instrument removal and re-instrumentation [3]. Since the overall recurrence rate in patients with pyogenic spondylodiscitis ranges from 8% to 20.4% [3,4,5,6,7,8,9], having similar recurrence rates in patients who undergo early spinal instrumentation should receive attention, considering the economic burden as well as potential morbidity.

Nevertheless, no previous studies have investigated the recurrence rate or its risk factors specifically in groups of patients who underwent early spinal instrumentation for pyogenic spondylodiscitis. Generally, most patients with pyogenic spondylodiscitis are treated conservatively with antibiotics. A small proportion of patients undergo surgical treatment, and only a fraction of them undergo surgery using spinal instruments early after the diagnosis. Since the reported yearly incidence of native pyogenic spondylodiscitis ranges between 2.2 to 4.8 per 100,000 [10,11,12,13], the risk factors for recurrence in patients who undergo early spinal instrumentation for pyogenic spondylodiscitis cannot be accurately evaluated due to the small sample size, even in multicenter studies. Therefore, studies demonstrating the safety of early spinal instrumentation only included several dozen patients among several hundreds of the entire cohort [1,2,3,4,5]. These limitations in patient sampling have resulted in an evaluation of recurrence risk factors in heterogenous cohorts including all patients regardless of treatment method, instead of a homogenous cohort of patients who underwent early spinal instrumentation.

Recurrence prediction models that are not based on a homogenous cohort of patients who underwent early spinal instrumentation have theoretical limitations for clinical use. Undrained abscesses, comorbid medical conditions, and resistant causative organisms are the three major risk factors for recurrence in patients with pyogenic spondylodiscitis [2,3,4,5,7,9]. However, early spinal instrumentation is the most aggressive form of treatment and is generally performed in healthy patients who can endure long-term surgery under general anesthesia. In addition, spine surgeons can sufficiently remove devitalized tissues, including abscesses, through firm stabilization. Therefore, among the three recurrence factors, preoperative undrained abscess and medical comorbidities have limited value in predicting recurrence in these patients. Instead, precise surgical approaches, type of instrumentation, or transfusion, which are generally known to be risk factors for postoperative infection after instrumented spinal surgery [14], should be additionally considered for these patients.

This study aimed to investigate the recurrence rate and its associated factors in patients who underwent early instrumented spinal fusion surgery for pyogenic spondylodiscitis. To overcome the limitations mentioned earlier, we used a domestic national claims database covering the entire population.

## 2. Methods

### 2.1. Database

In this nationwide population-based cohort study, data were obtained from the Health Insurance Review and Assessment Service (HIRA) database. The HIRA database contains all inpatient and outpatient data from hospitals and community clinics in Korea, allowing a nationwide cohort study. Diagnostic codes were assigned according to the modified version of the tenth revision of the International Classification of Diseases (ICD-10) and the seventh revision of the Korean Classification of Diseases (KCD-7). Drug use under-diagnosis was identified using anatomical therapeutic chemical codes and the HIRA general name codes. This study was approved by the Institutional Review Board of our hospital (IRB No. 2020-03-009-001).

### 2.2. Study Patients

We included patients aged >19 years who underwent early instrumented spinal fusion surgery for pyogenic spondylodiscitis between 1 January 2014, and 31 December 2018. Pyogenic spondylodiscitis (index disease) was identified using the following codes: intraspinal abscess (G06.1), osteomyelitis of the vertebra (M46.2), discitis (M46.3, M46.4), other/unspecified infection (M46.5, M46.8, M46.9, M49.2, M49.3), unspecified extradural and subdural abscess (G06.2), and infection following a procedure (T814). To guarantee the presence of pyogenic infection and completeness of the initial treatment for pyogenic spondylodiscitis according to international guidelines, we only included patients who were treated with antibiotics for over 6 weeks for the index disease [15]. Considering the mandatory 6-week antibiotic regimen according to international guidelines and the results from previous studies [4,5,7], early surgery (index surgery) was defined as spinal surgery performed within 6 weeks after the diagnosis of pyogenic spondylodiscitis.

We excluded patients who were treated under the ICD-10 codes of spinal infection or those who had undergone previous spinal surgeries within 2 years before the index disease. Patients with incomplete data were also excluded. A minimum follow-up period of one year was mandatory for study inclusion.

### 2.3. Outcomes: Recurrence after Early Spinal Instrumentation for Pyogenic Spondylodiscitis

**Figure 1:**
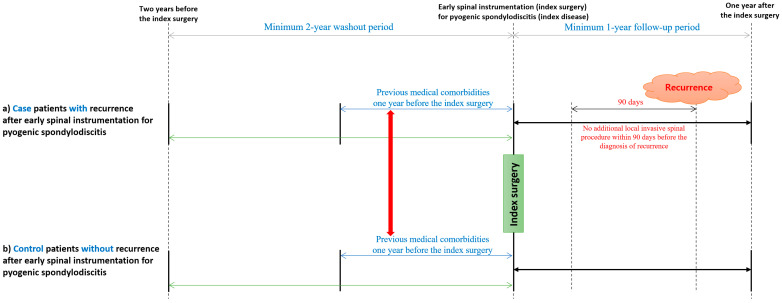
Definitions of cases and controls.

Recurrence (index recurrence) after early spinal instrumentation (index surgery) was initially defined for patients who were treated with antibiotics for more than 4 weeks under the main diagnosis of pyogenic spondylodiscitis after completion of the initial antibiotic treatment (>6 weeks) [7]. Recurrence was additionally defined on the basis of shorter (≥2 weeks) and longer (≥6 weeks) antibiotic treatment regimens after completion of initial antibiotic treatment, and sensitivity analysis was performed according to the two definitions. In addition, to exclude recurrences associated with additional spinal procedures after index surgery, we excluded patients who underwent additional local invasive spinal procedures within 90 days before the diagnosis of recurrence (Figure 1).

### 2.4. Data Collection Regarding Factors Associated with Recurrence

Intravenous and oral antibiotics were identified and classified using the codes listed in Supplementary Tables S1 and S2. Third- or fourth-generation cephalosporins, glycopeptides, carbapenems, and quinolones were categorized as antibiotics for resistant organisms. Preexisting medical comorbidities relevant to recurrence appearing within one year before the index surgery were identified according to ICD-10 codes (Supplementary Table S3) and evaluated on the basis of the Charlson comorbidity index (CCI). The CCI score is the sum of the weighted scores for each comorbidity and has shown good agreement with the ICD-10 codes [16,17]. Precise surgical approaches were identified and classified using the following electronic data interchange codes: anterior cervical approach (N2461, N0464, and N2463), posterior cervical approach (N2467, N2468, N0467, and N2469), anterior thoracic approach (N0465, N2464, N2465, and N2466), posterior thoracic approach (N0468), anterior lumbar approach (N0466, N1466), posterior lumbar approach (N0469, N1460, N1469, and N2470), and multiple approaches, including the two or more approaches mentioned. Data regarding transfusion (allogenous or autologous, Supplementary Table S4) and steroid use (Supplementary Table S5) during the index surgery were retrieved.

### 2.5. Statistical Analysis

Data are reported as mean ± standard deviation for numerical variables and number of patients and frequencies (%) for categorical variables. Logistic regression models were used to identify the factors associated with recurrence after early spinal instrumentation for pyogenic spondylodiscitis. All significant independent variables (*p* < 0.05) from the univariable analysis were included in the multivariable model. Multicollinearity between covariates was tested by using the variance inflation factor. The performance of the prediction model was assessed by the area under the receiver operating characteristic curve for discriminative ability and Hosmer–Lemeshow goodness-of-fit statistics for calibration.

Sensitivity analysis was performed according to the two different definitions for recurrence: shorter (2 weeks or more) and longer (6 weeks or more) durations of antibiotic therapy recurrence. We then assessed potential effect modification by unknown confounders by comparing the adjusted odds ratios (ORs) and 95% confidence intervals of the consistent risk factors that remained as significant predictors in the two different prediction models. The adjusted ORs were compared using the Bland and Altman interaction tests. The estimates of all statistical models were internally validated with relative bias based on 1000 bootstrapped samples. Data extraction and statistical analysis were performed using the SAS Enterprise Guide 6.1 (SAS Institute, Cary, NC, USA).

## 3. Results

**Figure 2:**
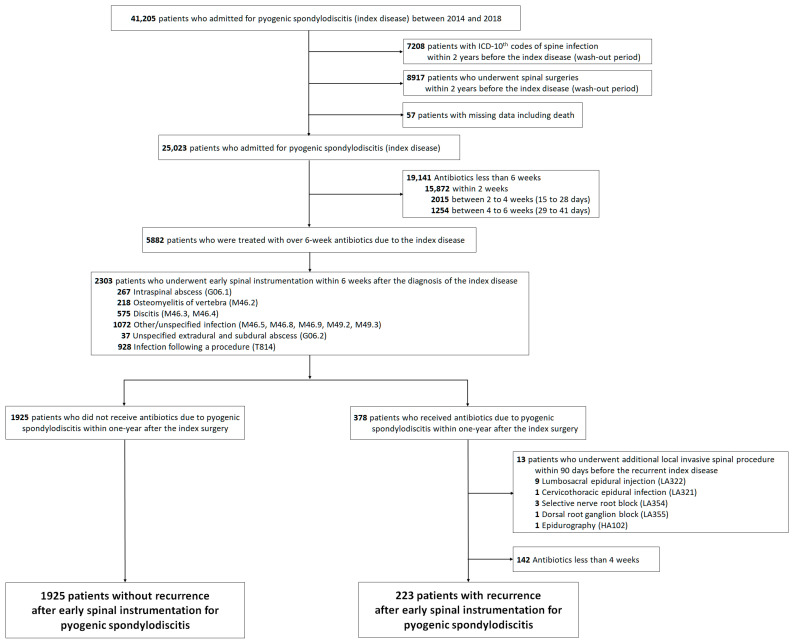
Selection of the study population.

Between 2014 and 2018, we identified 41,205 patients who were admitted with the ICD-10 codes for pyogenic spondylodiscitis (index disease, Figure 2). Among them, we excluded patients who were treated under the ICD-10 codes of spinal infection within 2 years before the index disease (*n* = 7208), those who had undergone spinal surgeries within 2 years before the index disease (*n* = 8917), and those who had missing data (*n* = 57). Among the remaining 25,023 patients, 19,141 received antibiotics for <6 weeks for the index disease, and they were excluded. Early spinal instrumentation was performed in 39% (*n* = 2303) of the remaining 5882 patients. Recurrence was identified in 378 patients, and among them, 13 patients who underwent additional local invasive spinal procedures within 90 days before recurrence and 142 patients who were treated with antibiotics for less than 4 weeks were excluded. Finally, 2148 patients were included in our study, including 1925 patients (90%) without recurrence and 378 patients (10%) with recurrence. The median age of the patients in the study cohort was 67 years (interquartile range, 58–74 years), and 52% (1110 of 2148) were men. The median interval between the index surgery and recurrence was 118 days (interquartile range, 100–175 days).

### 3.1. Incidence of Recurrence

**Figure 3:**
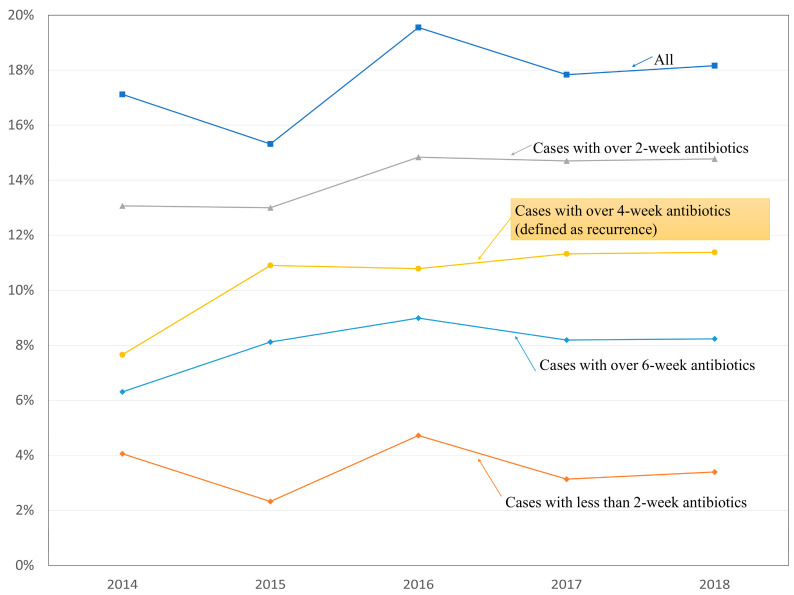
Recurrence rates based on the four definitions for pyogenic spondylodiscitis after early spinal instrumentation.

**Figure 4:**
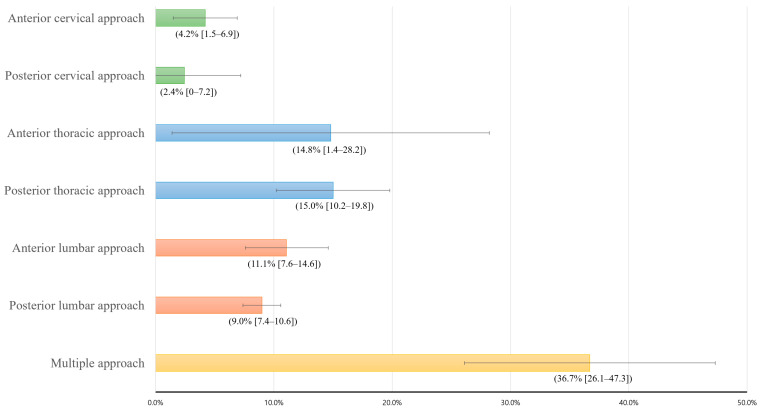
Recurrence rates according to the surgical procedures. The numbers in parentheses mean recurrence rates and their 95% confidence intervals.

The incidence of recurrence after early spinal instrumentation for pyogenic spondylodiscitis is presented in relation to the duration of antibiotic therapy (Table 1). The overall incidence of postoperative infection was 17.6% (378 of 2148), and it did not show an increasing or decreasing trend during the study period (Figure 3). The incidence of postoperative infection decreased as the duration of antibiotic treatment for postoperative infection increased: 14.1% for over 2 weeks of treatment, 10.4% for over 4 weeks of treatment, and 8.0% for over 6 weeks of treatment (Table 1 and Figure 3). Recurrence rates and their 95% CI are also presented according to the surgical procedures (Figure 4).

### 3.2. Comparison of the Two Cohorts: Univariable Analysis

The baseline patient characteristics in the two cohorts are presented in Table 2. Older age, rheumatologic disease, and osteoporosis were associated with an increased risk of recurrence after early spinal instrumentation for pyogenic spondylodiscitis. Other individual medical comorbidities and CCI scores were not associated with an increased risk of recurrence.

The most common method for early spinal instrumentation was the posterior lumbar approach (60%, 1153 of 2148; Table 3). In comparison with the posterior lumbar approach, the anterior cervical approach (OR = 0.44 [0.22–0.89]) was associated with a lower risk of recurrence, and multiple approaches (OR = 5.87 [3.57–9.64]) were associated with a higher risk of recurrence. In addition, cage use (OR = 1.82 [1.31–2.55]) and transfusion (OR: 3.61 [2.43–5.36]) were also associated with higher recurrence risk.

The most commonly used antibiotics were first-generation cephalosporins (26%, 554 of 2148), while the second most common type was glycopeptide or linezolid antibiotics (25%, 540 of 2148; Table 4). Antibiotics for resistant organisms were more frequently used in patients with recurrence (74% vs. 50%) in patients who received initial antibiotics for a significantly longer period (80 vs. 57 days). In addition, prolonged use (>2 weeks) of systemic steroids was also associated with an increased risk of recurrence (OR: 3.03 [1.92–4.78]).

### 3.3. Factors Associated with Recurrence after Early Spinal Instrumentation for Pyogenic Spondylodiscitis: Multivariable Analysis with Bootstrap Validation

Multivariable analysis identified the following variables as factors associated with recurrence (Table 5): age of 60–69 years (OR = 2.42 [1.01–5.81]), age over 70 years (OR = 2.29 [1.03–5.78]), posterior thoracic approach (OR = 1.85 [1.16–2.96]), multiple surgical approach (OR = 4.17 [2.44–7.14]), use of a cage (OR = 1.66 [1.12–2.47]), transfusion (OR = 2.55 [1.65–3.94]), antibiotic use for resistant organisms (OR = 2.55 [1.80–3.63]), and systemic steroid use over 2 weeks (OR = [2.27 (1.39–3.71]). Multicollinearity among covariates was low, and all variance inflation factors were less than 1.7. The Hosmer–Lemeshow goodness-of-fit test indicated good calibration (*p* = 0.689), and the area under the receiver operating curve was 0.813.

All significant independent variables (*p* < 0.05) from the univariable analysis were included in this multivariable model. Relative bias was estimated as the difference between the mean bootstrapped regression coefficient estimates, and the mean parameter estimates of the multivariable model divided by the mean parameter estimates of the multivariable model.

**Figure 5:**
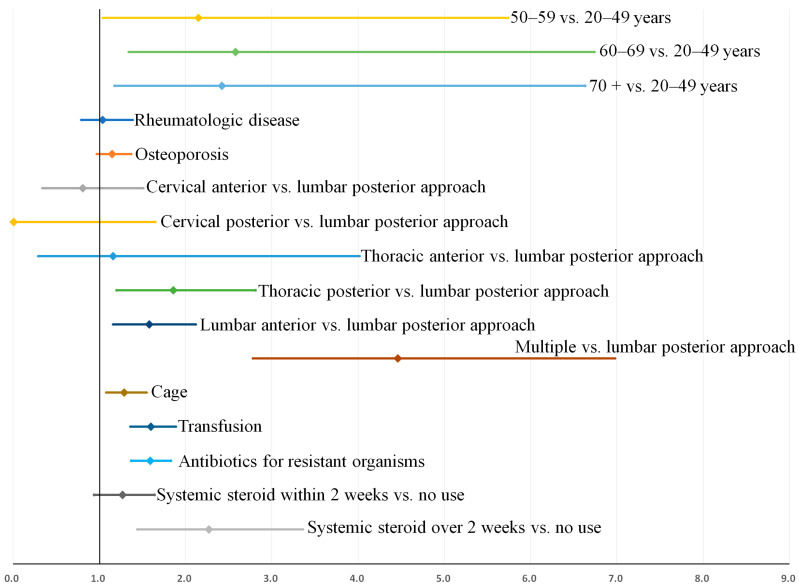
Factors associated with recurrence when recurrence was defined as an infection requiring over 4 weeks of antibiotic treatment: Bootstrap-adjusted odds ratios and their 95% confidence intervals (Table 5).

After bootstrap validation, the relative bias of most identified factors associated with recurrence was lower, between −0.2% and 6.9%, except for cage (−49.3%) and transfusion (−50.0%, Table 5). Bootstrap-adjusted odds ratios and 95% confidence intervals for the multivariable model are also displayed in Figure 5.

### 3.4. Sensitivity Analysis

**Figure 6:**
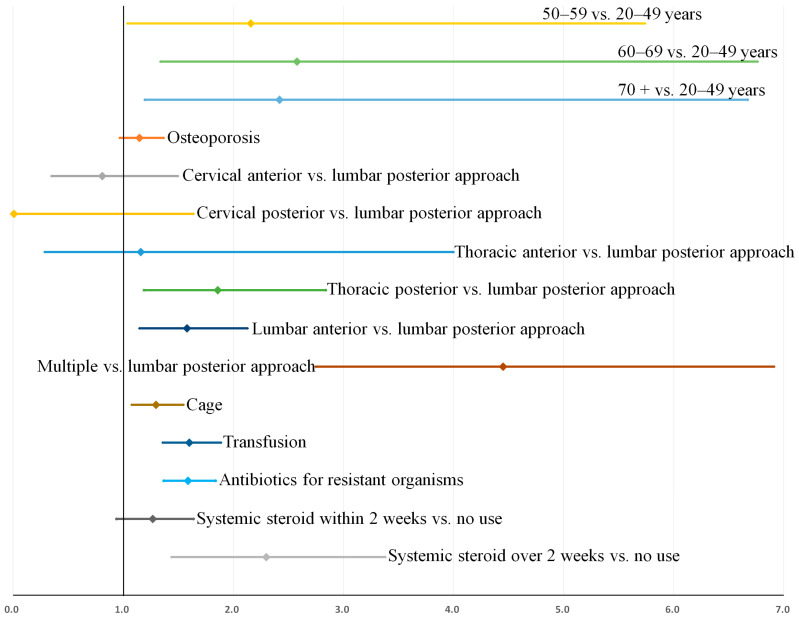
Factors associated with recurrence when recurrence was defined as an infection requiring over 2 weeks of antibiotic treatment: Bootstrap-adjusted odds ratios and their 95% confidence interval (Supplementary Table S6).

**Figure 7:**
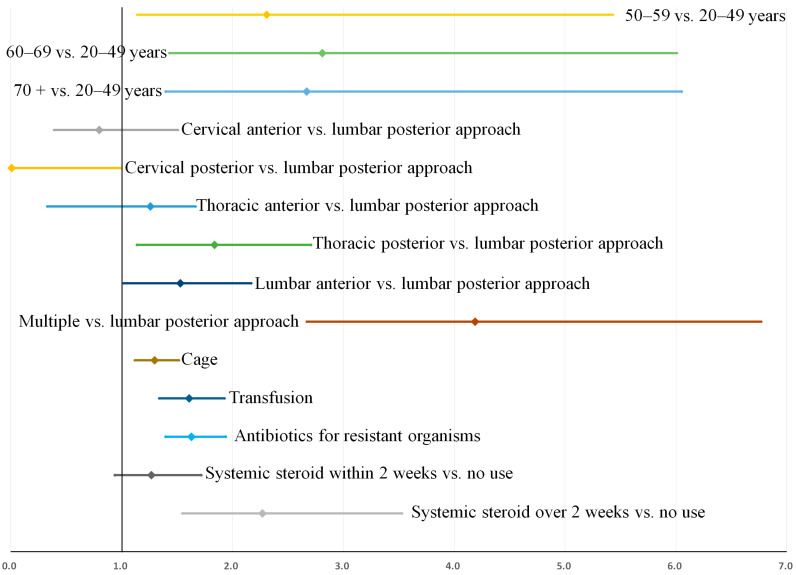
Associated factors for recurrence when recurrence was defined as an infection requiring over 6 weeks of antibiotic treatment: Bootstrap-adjusted odds ratios and their 95% confidence intervals (Supplementary Table S7).

A sensitivity analysis was performed to assess the influence of our definition of recurrence on our prediction model. When recurrence after early instrumentation for pyogenic spondylodiscitis was defined as recurrence requiring over 2 weeks of antibiotic treatment, 302 patients were identified as showing recurrence. Except for age >70 years, all associated factors in Table 5 remained consistently significant in this prediction model (Supplementary Table S6). When recurrence was defined as an infection requiring over 6 weeks of antibiotic treatment, 171 patients were identified as showing recurrence. All the associated factors in Table 5 also remained consistently significant in this prediction model (Supplementary Table S7). The bootstrap-adjusted odds ratios and confidence intervals for these two additional prediction models are shown in Figure 6 and Figure 7, respectively. Potential effect modification by unknown confounders was assessed by comparing the adjusted ORs and 95% confidence intervals of the consistent predictors in the two additional prediction models (models for recurrence requiring over 2 or 6 weeks of antibiotics), and are presented in Table 6. No significant effect modification was observed for any variable.

## 4. Discussion

Early spinal instrumentation for patients with pyogenic spondylodiscitis offers various clinical advantages, including preservation of vertebral structures, prevention of neurological deficits, and early ambulation. Therefore, assessment of the factors associated with recurrence is important for the appropriate use of this approach in patients with pyogenic spondylodiscitis. In this respect, our study, which was based on a nationwide cohort, is the largest study conducted for this purpose. Considering the paucity of studies with the same clinical premise, only two studies have separately reported the clinical outcomes of early spinal instrumentation for pyogenic spondylodiscitis, clearly defining the “early” period, and their reported recurrence rates ranged from 8% to 10% [4,5]. In our cohort, the recurrence rate with 2-, 4-, and 6-week antibiotic treatment regimens was 14.1% (302 of 2148), 10.4% (223 of 2148), and 8.0% (171 of 2148), respectively. Therefore, our recurrence rate data based on a nationwide database is consistent with the findings reported in previous studies.

On the basis of the findings for these 2148 homogenous patients who underwent early spinal instrumentation for pyogenic spondylodiscitis, we identified older age, surgical approaches including a posterior thoracic approach and multiple lumbar approaches, use of a cage, transfusion, antibiotic treatment for resistant organisms, and systemic steroid treatment for over 2 weeks as factors associated with recurrence of infection (Table 5 and Figure 3). Among the three major recurrence risk factors for pyogenic spondylodiscitis, including undrained abscess, comorbid medical condition, and resistant causative organisms [2,3,4,5,7,9], comorbid medical conditions were not factors associated with recurrence in our patients (Table 5). Our study, which was based on a homogenous group of patients, enabled us to evaluate surgical factors associated with recurrence, and among the surgical factors, we especially focused on surgical approaches for early spinal instrumentation (Figure 4 and Table 3). Postoperative infection after instrumented spinal surgery for degenerative diseases is usually less frequent in the cervical spine than in other spinal regions [14]. However, in our cohort of patients with pyogenic spondylodiscitis, multivariable analysis did not show a significantly lower postoperative infection rate (recurrence) after early cervical spinal instrumentation (Table 5). Instead, the recurrence risk was significantly higher in patients who underwent posterior thoracic or multiple spinal instrumentations.

Multiple spinal instrumentations were the most prominent factor associated with recurrence in our recurrence prediction model (OR = 4.17 [2.44–7.14], Table 5). In addition, cage use (OR = 1.66 [1.12–2.47]) and transfusion (OR = 2.55 [1.65–3.94]) during the surgical treatment were also associated with a higher recurrence risk. These results are interesting because these three surgical factors, which represent surgical invasiveness, have been regarded as risk factors for postoperative infection in patients who underwent surgery for non-infectious spinal disease [18,19]. With developments in protocols for medico-surgical treatment of pyogenic spondylodiscitis [15], recent studies have consistently reported that early spinal instrumentation for pyogenic spondylodiscitis yields favorable clinical outcomes in terms of recurrence and mortality [3,4,5,7]. In these studies, although insertion of a metal instrument during active infection theoretically had a negative influence on infection control, such early spinal instrumentation was not associated with increased recurrence risk, and sufficient removal of the abscess through wide decompressive surgery was considered as a possible cause. However, our results suggest that although early spinal instrumentation is safe for patients with spondylodiscitis in terms of recurrence, it should be carefully chosen considering the factors associated with recurrence (Table 5) and performed using a minimal spinal instrument required to stabilize the vertebral structure after abscess removal. The cause of the higher recurrence risk in the posterior thoracic approach cannot be identified in this study, and we propose that the posterior thoracic approach offers limited accessibility to abscesses generally located anterior to the thoracic spinal cord, increasing the risk of recurrence associated with an insufficient abscess removal.

The main strength of our study using a nationwide database is that we included a sufficient number of homogenous patients (*n* = 2148) who underwent early spinal instrumentation for pyogenic spondylodiscitis. Although numerous studies have evaluated patients with pyogenic spondylodiscitis, none of them, to our knowledge, have demonstrated the recurrence rate and its risk factors in a large, homogenous patient population. In addition, we attempted to minimize the influence of unknown confounders on our results by using a two-step validation procedure. First, we performed an internal validation using bootstrap-adjusted logistic regression analysis. Bootstrapping is a sampling method that uses random sampling with replacements. The confidence intervals calculated from bootstrap sampling (1000 times in our study) are asymptotically more accurate than those obtained using sample variance based on assumptions of normality. Bootstrapping can reduce the possible skewness of independent variables, including unknown confounders. Second, we performed a sensitivity analysis based on the three definitions of recurrence according to the duration of antibiotic treatment for recurrent infection, and our results were consistent regardless of the definitions.

Despite these strengths, our results should be interpreted with caution considering the following limitations. First, the HIRA database is a national claims database and was not originally designed for clinical research. Possible discrepancies between the diagnostic codes in the database and the actual diseases can be potential sources of bias [20]. On the other hand, the HIRA system is based on the compulsory national health insurance system, and government officials thoroughly review all claims data in accordance with various regulatory protocols. Among them, the control policy for the use of antibiotics has been the object of priority control targets by government officials. Therefore, the information in the database for drug use, including antibiotic treatment and the use of costly procedural methods such as precise methods of spinal instrumentation or other invasive local spinal procedures, is very accurate. This information was the foundation of our study. Second, information related to possible risk factors such as precise surgical profiles, including differences in sterilization procedures, surgical protocols, and techniques among hospitals or surgeons, could not be included in the study, and the data for causative organisms were substituted with those for the use of antibiotics for resistant organisms. Although the consistency of our results was confirmed using two-step validation procedures, this approach may have led to bias. Third, we simply proposed that all patients received sufficient treatment for the index disease, only by the standard 6-week duration of antibiotic treatment. Although international guidelines based on randomized controlled trials suggest that 6-week antibiotic regimens are sufficient for native pyogenic spondylodiscitis [15,21], rare infections persisting even after 6-week antibiotic regimens could have led to bias.

In conclusion, our population-based study using the nationwide database is the largest clinical study based on a homogenous group of 2148 patients who underwent early spinal instrumentation for pyogenic spondylodiscitis. Recurrence was defined in detail according to the duration of antibiotic treatment regimens for recurrence, and the recurrence rate with 2-, 4-, and 6-week antibiotic treatment regimens was 14.1% (302 of 2148), 10.4% (223 of 2148), and 8.0% (171 of 2148), respectively. We also identified age, posterior thoracic approach, multiple surgical approaches, use of a cage, transfusion, antibiotics for resistant organisms, and systemic steroid treatment over 2 weeks as the factors associated with recurrence. The recurrence rates and associated factors can be practically used as a reasonable reference and provide clinicians with an acceptable tool for the comprehensive risk assessment for recurrence after early spinal instrumentation for pyogenic spondylodiscitis.

## Figures and Tables

**Table 1:** Incidence of recurrence according to duration of therapeutic antibiotics.

Year	Early Spinal Instrumentation (*n*)	Recurrence According to Duration of Therapeutic Antibiotics									
		ALL		Less than 2 Weeks		Over 2 Weeks (Included in Sensitivity Analysis)		Over 4 Weeks (Defined as Index Recurrence)		Over 6 Weeks (Included in Sensitivity Analysis)	
		(*n*)	Incidence (%)	(*n*)	Incidence (%)	(*n*)	Incidence (%)	(*n*)	Incidence (%)	(*n*)	Incidence (%)
2014	444	76	17.1%	18	4.1%	58	13.1%	34	7.7%	28	6.3%
2015	431	66	15.3%	10	2.3%	56	13.0%	47	10.9%	35	8.1%
2016	445	87	19.6%	21	4.7%	66	14.8%	48	10.8%	40	9.0%
2017	415	74	17.8%	13	3.1%	61	14.7%	47	11.3%	34	8.2%
2018	413	75	18.2%	14	3.4%	61	14.8%	47	11.4%	34	8.2%
All *	2148	378	17.6% (16.0–19.2)	76	3.5% (2.8–4.3)	302	14.1% (12.6–15.5)	223	10.4% (9.1–11.7)	171	8.0% (6.8–9.1)

* Incidence was presented with rates and 95% confidence interval.

**Table 2:** Comparison of baseline patient characteristics.

Variables	Category	Patients without Recurrence (*n* = 1925)	Patients with Recurrence (*n* = 223)	Base Infection Rates (%)	Odds Ratio	*p*-Value
Age	Median with interquartile range	67 (58–73)	70 (62–75)			<0.001
	20–49	168 (9)	6 (3)	3.4%	reference	
	50–59	386 (20)	30 (13)	7.2%	2.18 (0.89–5.33)	0.089
	60–69	585 (30)	72 (32)	11.0%	3.45 (1.47–8.07)	0.004
	70+	786 (41)	115 (52)	12.8%	4.10 (1.77–9.47)	0.001
Sex	Male	991 (51)	122 (55)	11.0%	1.14 (0.86–1.50)	0.361
	Female	934 (49)	101 (45)	9.8%	reference	
Region of residence	Urban	1548 (80)	177 (79)	10.3%	reference	
	Rural	377 (20)	46 (21)	10.9%	1.07 (0.76–1.50)	0.711
Type of hospital	Tertiary	694 (36)	95 (43)	12.0%	1.39 (0.98–1.98)	0.067
	General hospital	683 (35)	74 (33)	9.8%	1.10 (0.76–1.59)	0.614
	Others	548 (28)	54 (24)	9.0%	reference	0.142
Charlson comorbidity index score	Mean ± SD	2.42 ± 2.12	2.28 ± 2.22			
	0–2	1152 (60)	128 (57)	10.0%	reference	
	3–5	587 (30)	73 (33)	11.1%	1.12 (0.83–1.52)	0.640
	≥6	186 (10)	22 (10)	10.6%	1.07 (0.66–1.72)	0.979
Comorbidities	Myocardial infarction	24 (1)	4 (2)	14.3%	1.45 (0.50–4.21)	0.496
	Congestive heart failure	102 (5)	15 (7)	12.8%	1.29 (0.74–2.26)	0.375
	Peripheral vascular disease	275 (14)	34 (15)	11.0%	1.08 (0.73–1.59)	0.699
	Cerebrovascular disease	233 (12)	35 (16)	13.1%	1.35 (0.92–1.99)	0.125
	Dementia	40 (2)	5 (2)	11.1%	1.08 (0.42–2.77)	0.871
	Chronic pulmonary disease	508 (26)	61 (27)	10.7%	1.05 (0.77–1.43)	0.757
	Rheumatologic disease	80 (4)	17 (8)	17.5%	1.90 (1.11–3.28)	0.020
	Peptic ulcer disease	364 (19)	50 (22)	12.1%	1.24 (0.89–1.73)	0.209
	Liver disease					
	Mild	175 (9)	22 (10)	11.2%	1.10 (0.69–1.75)	0.705
	Moderate to severe	10 (1)	1 (0)	9.1%	0.86 (0.11–6.77)	0.888
	Diabetes					
	Uncomplicated	571 (30)	60 (27)	9.5%	0.87 (0.64–1.19)	0.393
	Complicated	196 (10)	19 (9)	8.8%	0.82 (0.50–1.35)	0.435
	Hemiplegia or paraplegia	42 (2)	4 (2)	8.7%	0.86 (0.31–2.42)	0.777
	Renal disease	93 (5)	12 (5)	11.4%	1.12 (0.60–2.08)	0.719
	End stage renal disease	45 (2)	5 (2)	10.0%	0.96 0.38–2.44)	0.930
	Malignancy					
	Primary (including lym phoma and leukemia)	139 (7)	19 (9)	12.0%	1.20 (0.73–1.98)	0.482
	Metastatic	28 (1)	2 (1)	6.7%	0.61 (0.15–2.59)	0.506
	Osteoporosis	324 (17)	53 (24)	14.1%	1.54 (1.11–2.14)	0.010

**Table 3:** Comparison of surgical profiles during the index surgery.

Variables	Category	Patients without Recurrence (*n* = 1925)	Patients with Recurrence (*n* = 223)	Base Infection Rates (%)	Odds Ratio	*p*-Value
Method of early instrumentation	Cervical					
	Anterior	205 (11)	9 (4)	4.2%	0.44 (0.22–0.89)	0.005
	Posterior	40 (2)	1 (0)	2.4%	0.25 (0.03–1.86)	0.084
	Thoracic					
	Anterior	23 (1)	4 (2)	14.8%	1.76 (0.60–5.18)	0.384
	Posterior	181 (9)	32 (14)	15.0%	1.79 (1.17–2.73)	0.065
	Lumbar					
	Anterior	273 (14)	34 (15)	11.1%	1.26 (0.84–1.89)	0.691
	Posterior	1153 (60)	114 (51)	9.0%	reference	
	Multiple approach	50 (3)	29 (13)	36.7%	5.87 (3.57–9.64)	<0.001
Cage		281 (15)	53 (24)	15.9%	1.82 (1.31–2.55)	<0.001
Transfusion		1233 (64)	193 (87)	13.5%	3.61 (2.43–5.36)	<0.001

**Table 4:** Comparison of infection profiles during admission for the index disease.

Variables		Patients without Recurrence (*n* = 1925)	Patients with Recurrence (*n* = 223)	Base Infection Rates (%)	Odds Ratio	*p*-Value
Type of all used antibiotics	1st gen. cephalosporin	509 (26)	45 (20)			
	2nd gen. cephalosporin	375 (19)	25 (11)			
	3rd gen. cephalosporin	334 (17)	49 (22)			
	4th gen. cephalosporin	23 (1)	9 (4)			
	Other beta-lactams	259 (13)	51 (23)			
	Glycopeptide and linezolid	454 (24)	86 (39)			
	Carbapenem	72 (4)	23 (10)			
	Quinolone	228 (12)	64 (29)			
Antibiotics for resistant organisms *		960 (50)	166 (74)	14.7%	3.50 (2.50–4.90)	<0.001
Duration of initial antibiotics	Mean ± SD	57 ± 19	80 ± 17			<0.001
Use of systemic steroid	None	1231 (64)	119 (53)	8.8%	reference	
	Within 2 weeks	595 (31)	75 (34)	11.2%	1.30 (0.96–1.77)	0.087
	Over 2 weeks	99 (5)	29 (13)	22.7%	3.03 (1.92–4.78)	<0.001

* Third- or fourth-generation cephalosporins, glycopeptides, linezolid, carbapenems, and quinolones were categorized as antibiotics for resistant organisms.

**Table 5:** Factors associated with recurrence: multivariable analysis with bootstrap validation.

Variables	Category	Adjusted Odds Ratio (95% Confidence Interval)	*p*-Value	Bootstrap Adjusted Odds Ratio (95% Confidence Interval)	Relative Bias (%)
Age	50–59 vs. 20–49	2.07 (0.83–5.16)	0.118	2.15 (1.04–5.74)	5.5
	60–69 vs. 20–49	2.42 (1.01–5.81)	0.048	2.58 (1.34–6.74)	7.3
	70+ vs. 20–49	2.29 (1.03–5.78)	0.044	2.42 (1.18–6.63)	6.9
Comorbidities	Rheumatologic disease	1.13 (0.59–2.16)	0.716	1.04 (0.79–1.39)	−66.0
	Osteoporosis	1.33 (0.92–1.93)	0.129	1.15 (0.97–1.37)	−51.9
Method of early instrumentation	Cervical anterior vs. lumbar posterior	0.86 (0.41–1.81)	0.689	0.81 (0.34–1.51)	35.8
	Cervical posterior vs. lumbar posterior	0.46 (0.06–3.45)	0.485	0.01 (0.00–1.65)	574.2
	Thoracic anterior vs. lumbar posterior	1.49 (0.48–4.60)	0.487	1.16 (0.29–4.02)	−62.1
	Thoracic posterior vs. lumbar posterior	1.85 (1.16–2.96)	0.010	1.86 (1.20–2.81)	1.2
	Lumbar anterior vs. lumbar posterior	1.55 (1.00–2.41)	0.052	1.58 (1.16–2.12)	3.8
	Multiple vs. lumbar posterior	4.17 (2.44–7.14)	<0.001	4.46 (2.78–6.98)	4.8
Cage		1.66 (1.12–2.47)	0.012	1.29 (1.08–1.55)	−49.3
Transfusion		2.55 (1.65–3.94)	<0.001	1.60 (1.36–1.89)	−50.0
Antibiotics for resistant organisms		2.55 (1.80–3.63)	<0.001	1.59 (1.37–1.83)	−50.3
Systemic steroid	Within 2 weeks vs. no use	1.26 (0.90–1.75)	0.173	1.27 (0.94–1.64)	3.0
	Over 2 weeks vs. no use	2.27 (1.39–3.71)	0.001	2.27 (1.44–3.36)	−0.2

**Table 6:** The assessment of potential effect modification by unknown confounders through comparison of adjusted odds ratios across the two different definitions for recurrence: shorter (≥2 weeks) and longer (≥6 weeks) durations of antibiotic therapy for recurrence.

Variables	Category	Recurrence Defined by Over 2-Week Antibiotics	Recurrence Defined by Over 6-Week Antibiotics	*p*-Value (Bland–Altman Test for Interaction)
		Odds Ratio (95% CI)	Odds Ratio (95% CI)	
Age	50–59 vs. 20–49	2.07 (0.83–5.17)	2.01 (0.84–5.23)	0.964
	60–69 vs. 20–49	2.43 (1.01–5.83)	2.53 (1.06–6.07)	0.949
	70 + vs. 20–49	2.29 (0.96–5.47)	2.47 (1.04–5.86)	0.904
Method of early instrumentation	Cervical anterior vs. lumbar posterior	0.86 (0.41–1.81)	0.85 (0.41–1.80)	0.983
	Cervical posterior vs. lumbar posterior	0.49 (0.06–3.67)	0.47 (0.06–3.53)	0.978
	Thoracic anterior vs. lumbar posterior	1.50 (0.48–4.62)	1.51 (0.49–4.67)	0.994
	Thoracic posterior vs. lumbar posterior	1.85 (1.56–2.96)	1.84 (1.15–2.94)	0.985
	Lumbar anterior vs. lumbar posterior	1.55 (1.00–2.41)	1.55 (1.00–2.41)	1.000
	Multiple vs. lumbar posterior	4.16 (2.43–7.12)	4.13 (2.41- 7.07)	0.985
Cage		1.67 (1.12–2.48)	1.68 (1.13–2.49)	0.983
Transfusion		2.55 (1.65–3.94)	2.52 (1.64–3.89)	0.970
Antibiotics for resistant organisms		2.55 (1.80–3.63)	2.55 (1.80–3.62)	1.000
Systemic steroid	Within 2 weeks vs. no use	1.26 (0.90–1.75)	1.26 (0.91–1.75)	1.000
	Over 2 weeks vs. no use	2.30 (1.42–3.74)	2.29 (1.41–3.71)	0.990

## Data Availability

The datasets generated for the current study are not publicly available due to Data Protection Laws and Regulations in Korea, but the analyzing results are available from the corresponding authors on reasonable request.

## Supplementary Materials

The following supporting information can be downloaded at: https://www.mdpi.com/article/10.3390/jcm11123356/s1, Table S1: HIRA general name codes for intravenous antibiotics; Table S2: HIRA general name codes for oral antibiotics; Table S3: ICD-10 codes for comorbidities including Charlson comorbidities index items and scores; Table S4: HIRA therapeutic codes for transfusion; Table S5: HIRA general name codes for used steroids; Table S6: Risk factors for recurrence when recurrence was defined as an infection requiring over 2-week antibiotics; Table S7: Risk factors for recurrence when recurrence was defined as an infection requiring over 6-week antibiotics.
