# Supplementary material for: Recurrence Rates and Its Associated Factors after Early Spinal Instrumentation for Pyogenic Spondylodiscitis: A Nationwide Cohort Study of 2148 Patients
Source: J Clin Med. 2022 Jun 11;11(12):3356. doi: 10.3390/jcm11123356 (PMC9225581; doi:10.3390/jcm11123356)
Supplement: Supplementary file 1 [file jcm-11-03356-s001.zip › jcm-1726672-supplementary.pdf]

Supplementary Table S1. HIRA general name codes for intravenous antibiotics

Category of antibiotics	Type of antibiotics	ATC code	HIRA general name code
1st gen. cephalosporin	cefazolin sodium 1g	J01DB04	125701BIJ
	ceftezole sodium 1g	J01DB12	128501BIJ
	ceftezole sodium 2g	J01DB12	128502BIJ
	ceftezole sodium 0.5g	J01DB12	128503BIJ
	cefazedone sodium 1g	J01DB06	130001BIJ
	cefazedone sodium 2g	J01DB06	130002BIJ
	cefazedone sodium 0.5g	J01DB06	130003BIJ
	cefradine 1g	J01DB09	130102BIJ
2nd gen. cephalosporin	cefamandole nafate 1g	J01DC03	125401BIJ
	cefmetazole sodium 1g	J01DC09	126501BIJ
	cefmetazole sodium 0.5g	J01DC09	126502BIJ
	cefmetazole sodium 2g	J01DC09	126503BIJ
	cefmetazole sodium 1g	J01DC09	468100BIJ
	cefbuperazone sodium 1g	J01DC13	125801BIJ
	cefbuperazone sodium 0.5g	J01DC13	125802BIJ
	cefminox sodium 1g	J01DC12	126601BIJ
	cefotetan disodium (as cefotetan 1g)	J01DC05	127201BIJ
	cefotetan disodium 1g	J01DC05	482900BIJ
	cefotiam hydrochloride 1g	J01DC07	127301BIJ
	cefotiam hydrochloride 0.5g	J01DC07	127302BIJ
	cefotiam hydrochloride 0.3mg	J01DC07	127303BIJ
	cefotiam hydrochloride 1g	J01DC07	465400BIJ
	cefuroxime sodium 1.5g	J01DC02	129001BIJ
	cefuroxime sodium 0.75g	J01DC02	129003BIJ
	flomoxef sodium 0.5g	J01DC14	159501BIJ
3rd gen. cephalosporin	ceftazidime hydrate 1g	J01DD02	128301BIJ
	ceftazidime hydrate 0.5g	J01DD02	128302BIJ
	ceftazidime hydrate 2g	J01DD02	128303BIJ
	cefodizime sodium 1g	J01DD09	126701BIJ

	cefodizime sodium 0.5g	J01DD09	126702BIJ
	cefotaxime sodium 1g	J01DD01	127101BIJ
	cefotaxime sodium 2g	J01DD01	127102BIJ
	cefotaxime sodium 0.5g	J01DD01	127103BIJ
	cefoxitin sodium 1g	J01DC01	127501BIJ
	cefpiramide sodium 1g	J01DD11	127701BIJ
	cefpiramide sodium 0.5g	J01DD11	127702BIJ
	cefpirome sulfate 1g	J01DE02	127801BIJ
	ceftizoxime sodium 1g	J01DD07	128701BIJ
	ceftriaxone sodium hydrate 1g	J01DD04	128801BIJ
	ceftriaxone sodium hydrate 0.25g	J01DD04	128802BIJ
	ceftriaxone sodium hydrate 2g	J01DD04	128803BIJ
	ceftriaxone sodium hydrate 0.5g	J01DD04	128804BIJ
	ceftriaxone sodium hydrate 1.5g	J01DD04	128805BIJ
	ceftriaxone sodium 1g	J01DD04	468200BIJ
	ceftriaxone sodium 2g	J01DD04	478500BIJ
	cefoperazone sodium 0.5g	J01DD12	329900BIJ
	cefoperazone sodium 1g	J01DD12	557400BIJ
	cefoperazone sodium 0.5g	J01DD12	463100BIJ
4th gen. cephalosporin	cefepime hydrochloride hydrate 1g	J01DE01	126101BIJ
	cefepime hydrochloride hydrate 0.5g	J01DE01	126103BIJ
	cefepime hydrochloride hydrate·L-arginine (as cefepime hydrochloride 1g)	J01DE01	800001BIJ
Other beta-lactams	benzathine penicillin G 120M unit	J01CE08	115502BIJ
	penicillin G potassium crystal 5MI.U	J01CE01	210001BIJ
	piperacillin sodium 2g tazobactam 0.25g	J01CR05	329500BIJ
	piperacillin sodium 4g tazobactam 0.5g	J01CR05	329600BIJ
	amoxicillin sodium 0.5g clavulanate potassium 0.1g	J01CR02	328900BIJ
	amoxicillin sodium 1g clavulanate potassium 0.2g	J01CR02	329000BIJ
	amoxicillin sodium (as amoxicillin 1g)	J01CA04	108201BIJ
	amoxicillin sodium (as amoxicillin 0.5g)	J01CA04	108203BIJ
	ampicillin sodium 0.5g	J01CA01	108603BIJ

	nafcillin sodium 1g	J01CF06	360801BIJ
	amoxicillin sodium 1g sulbactam sodium 0.5g	J01CR02	381300BIJ
	amoxicillin sodium 0.5g sulbactam sodium 0.25g	J01CR02	381500BIJ
	ampicillin sodium 0.5g sulbactam sodium 0.25g	J01CR01	328500BIJ
	ampicillin sodium 1g sulbactam sodium 0.5g	J01CR01	328600BIJ
	piperacillin sodium 1g sulbactam sodium 0.5g	J01CR05	433300BIJ
	piperacillin sodium 2g sulbactam sodium 1g	J01CR05	453200BIJ
	piperacillin sodium 3g sulbactam sodium 1.5g	J01CR05	657600BIJ
Glycopeptide and linezolid	teicoplanin 0.2g	J01XA02	234901BIJ
	teicoplanin 0.4g	J01XA02	234902BIJ
	teicoplanin 0.2g	J01XA02	501000BIJ
	vancomycin hydrochloride 1g	J01XA01	247203BIJ
	vancomycin hydrochloride 0.25g	J01XA01	247204BIJ
	vancomycin hydrochloride 0.5g	J01XA01	247205BIJ
	linezolid 0.6g(2mg/mL)	J01XX08	412930BIJ
	vancomycin hydrochloride 1g	J01XA01	479800BIJ
Lincosamide	lincomycin hydrochloride 0.3g(0.3g/mL)	J01FF02	184230BIJ
	lincomycin hydrochloride 0.6g(0.3g/mL)	J01FF02	184231BIJ
	clindamycin phosphate 0.3g(0.15g/mL)	J01FF01	135630BIJ
	clindamycin phosphate 0.6g(0.15g/mL)	J01FF01	135631BIJ
Macrolide	amikacin sulfate 0.25g(0.125g/mL)	J01GB06	106831BIJ
	amikacin sulfate 0.5g(0.25g/mL)	J01GB06	106833BIJ
	amikacin sulfate 0.5g(5mg/mL)	J01GB06	106834BIJ
	tobramycin 80mg(40mg/mL)	J01GB01	240841BIJ
	tobramycin 0.1g(50mg/mL)	J01GB01	240843BIJ
	azithromycin hydrate (as azithromycin 0.5g)	J01FA10	112734BIJ
	gentamicin sulfate 80mg(40mg/mL)	J01GB03	165136BIJ
	isepamicin sulfate 0.2g(0.1g/mL)	J01GB11	177730BIJ
	netilmicin sulfate 0.15g(0.1g/mL)	J01GB07	200732BIJ
	netilmicin sulfate 50mg(25mg/mL)	J01GB07	200730BIJ
	netilmicin sulfate 0.1g(50mg/mL)	J01GB07	200731BIJ

	netilmicin sulfate 0.15g(75mg/mL)	J01GB07	200733BIJ
	ribostamycin sulfate 0.5g(0.3333g/mL)	J01GB10	223830BIJ
	ribostamycin sulfate 1g(0.3333g/mL)	J01GB10	223831BIJ
	ribostamycin sulfate 1g	J01GB10	223801BIJ
	ribostamycin sulfate 0.5g	J01GB10	223802BIJ
	spectinomycin hydrochloride 2g	J01XX04	230801BIJ
	arbekacin sulfate 75mg(50mg/mL)	J01GB12	360930BIJ
	arbekacin sulfate 0.1g(50mg/mL)	J01GB12	360931BIJ
Carbapenem	meropenem 0.5g	J01DH02	190702BIJ
	meropenem 1g	J01DH02	190703BIJ
	meropenem 2g	J01DH02	190704BIJ
	imipenem hydrate (as imipenem 0.25g)	J01DH51	329300BIJ
	imipenem hydrate (as imipenem 0.5g)	J01DH51	329400BIJ
	imipenem monohydrate 0.5g	J01DH51	466100BIJ
	ertapenem sodium (as ertapenem 1g)	J01DH03	447701BIJ
	doripenem monohydrate (as doripenem 0.25g)	J01DH04	593201BIJ
Quinolone	ciprofloxacin 0.1g(2mg/mL)	J01MA02	134133BIJ
	ciprofloxacin 0.2g(2mg/mL)	J01MA02	134134BIJ
	ciprofloxacin 0.4g(2mg/mL)	J01MA02	134135BIJ
	levofloxacin 0.25g(5mg/mL)	J01MA12	183233BIJ
	levofloxacin 0.5g(5mg/mL)	J01MA12	183234BIJ
	levofloxacin 0.75g(5mg/mL)	J01MA12	183235BIJ
	levofloxacin 1g(5mg/mL)	J01MA12	183236BIJ
	ofloxacin 0.2g(2mg/mL)	J01MA01	203940BIJ
	moxifloxacin hydrochloride (as moxifloxacin 0.4g(1.6mg/mL))	J01MA14	380335BIJ
	gemifloxacin mesylate (as gemifloxacin 0.2g)	J01MA15	442902BIJ
	moxifloxacin 0.4g(1.6mg/mL)	J01MA14	801601BIJ
ETC	aztreonam 1g	J01DF01	113001BIJ
	aztreonam 0.5g	J01DF01	113002BIJ
	colistin sodium methanesulfonate 0.15g	J01XB01	484201BIJ
	colistin sodium methanesulfonate 0.16g	J01XB01	484203BIJ

	sulfamethoxazole 0.4g(80mg/mL) trimethoprim 80mg(16mg/mL)		J01EE01	330000BIJ

Supplementary Table S2. HIRA general name codes for oral antibiotics

Category of antibiotics	Type of antibiotics	ATC code	HIRA general name code
1st gen. cephalosporin	cefadroxil 11.75g(25mg/mL)	J01DB05	125333ASY
	cefadroxil 7.5g(50mg/mL)	J01DB05	125332ASY
	cefadroxil 23.5g(50mg/mL)	J01DB05	125334ASY
	cefadroxil hydrate 0.5g	J01DB05	125304ACH
	cefroxadine 0.25g	J01DB11	128101ACH
	cephalexin 0.5g	J01DB01	129402ACH
	methylol cephalexin lysinate 0.5g	J01DB01	193101ACH
	methylol cephalexin lysinate 0.5g	J01DB01	193101ATB
	cefradine hydrate 0.25g	J01DB09	130101ACH
	cefradine hydrate 0.5g	J01DB09	130105ACH
2nd gen. cephalosporin	cefaclor hydrate 0.75g(25mg/mL)	J01DC04	125232ASY
	cefaclor hydrate 2.5g(25mg/mL)	J01DC04	125235ASY
	cefaclor hydrate 3.75g(25mg/mL)	J01DC04	125237ASY
	cefaclor hydrate 5g(25mg/mL)	J01DC04	125238ASY
	cefaclor hydrate 0.25g	J01DC04	125201ACH
	cefaclor hydrate 0.375g	J01DC04	125204ATR
	cefprozil 1.5g(25mg/mL)	J01DC10	128030ASY
	cefprozil 2.5g(25mg/mL)	J01DC10	128031ASY
	cefprozil 3.75g(25mg/mL)	J01DC10	128032ASY
	cefprozil hydrate 0.25g	J01DC10	128001ATB
	cefuroxime axetil (as cefuroxim 1.25g(25mg/mL))	J01DC02	128931ASY
	cefuroxime axetil (as cefuroxime 0.25g)	J01DC02	128903ATB
3rd gen. cephalosporin	ceftibuten 0.2g	J01DD14	128602ACH
	cefdinir 10g(0.1g/g)	J01DD15	125931AGN
	cefdinir 0.1g	J01DD15	125901ACH
	cefditoren pivoxil 10g(0.1g/g)	J01DD16	126031AGN
	cefditoren pivoxil 0.1g	J01DD16	126001ATB
	cefetamet pivoxil hydrochloride 0.5g	J01DD10	126201ATB
	cefixime hydrate 5g(50mg/g)	J01DD08	126334APD

	cefixime hydrate 0.1g	J01DD08	126301ACH
	cefpodoxime proxetil 0.5g(10mg/mL)	J01DD13	127930ASY
	cefpodoxime proxetil 2g(10mg/mL)	J01DD13	127931ASY
	cefpodoxime proxetil 5g(10mg/mL)	J01DD13	127932ASY
	cefpodoxime proxetil 0.1g	J01DD13	127901ATB
	cefcapene pivoxil hydrochloride hydrate 10g(0.1g/g)	J01DD17	474432AGN
	cefcapene pivoxil hydrochloride hydrate 75mg	J01DD17	474401ATB
	cefcapene pivoxil hydrochloride hydrate 0.1g	J01DD17	474402ATB
Other beta-lactams	sultamicillin tosylate (as sultamicillin 0.375g)	J01CR04	233701ATB
	amoxicillin 6g(0.12g/mL) clavulanate potassium 0.429g(8.58mg/mL)	J01CR02	536300ASY
	amoxicillin 60g(0.12g/mL) clavulanate potassium 4.29g(8.58mg/mL)	J01CR02	647300ASY
	amoxicillin 750mg(25mg/mL) clavulanate potassium 0.1875g(6.25mg/mL)	J01CR02	534200ASY
	amoxicillin 1.25g(25mg/mL) clavulanate potassium 0.3125g(6.25mg/mL)	J01CR02	534400ASY
	amoxicillin 6.25g(25mg/mL) clavulanate potassium 1.5625g(6.25mg/mL)	J01CR02	534600ASY
	amoxicillin 12.5g(25mg/mL) clavulanate potassium 3.125g(6.25mg/mL)	J01CR02	534700ASY
	amoxicillin 1.2g(40mg/mL) clavulanate potassium 0.171g(5.7mg/mL)	J01CR02	534800ASY
	amoxicillin 2g(40mg/mL) clavulanate potassium 0.285g(5.7mg/mL)	J01CR02	535000ASY
	amoxicillin 10g(40mg/mL) clavulanate potassium 1.425g(5.7mg/mL)	J01CR02	535300ASY
	amoxicillin 20g(40mg/mL) clavulanate potassium 2.85g(5.7mg/mL)	J01CR02	535500ASY
	amoxicillin 0.125g clavulanate potassium 62.5mg	J01CR02	310500ATB
	amoxicillin 0.25g clavulanate potassium 0.125g	J01CR02	310600ATB
	amoxicillin 0.5g clavulanate potassium 0.125g	J01CR02	310700ATB
	amoxicillin 0.875g clavulanate potassium 0.125g	J01CR02	440100ATB
	amoxicillin 0.4375g clavulanate potassium 62.5mg	J01CR02	462000ATB
	amoxicillin 0.125g clavulanate potassium 31.25mg	J01CR02	467300ASS
	amoxicillin 0.2g clavulanate potassium 50mg	J01CR02	467400ASS
	amoxicillin 12.5g(25mg/mL)	J01CA01	108130ASY
	amoxicillin 0.25g	J01CA01	108101ACH
	amoxicillin 0.5g	J01CA01	108103ACH
	amoxicillin 3g(50mg/mL) pivoxil sulbactam 3g(50mg/mL)	J01CR02	536200ASY

	amoxicillin 0.25g pivoxil sulbactam 0.25g	J01CR02	380000ATB
	ampicillin 0.25g	J01CR01	589301ACH
	ampicillin 0.5g	J01CR01	589302ACH
Glycopeptide and linezolid	vancomycin hydrochloride 0.25g	J01XA01	247202ACH
	linezolid 0.6g	J01XX08	412901ATB
	linezolid 0.3g	J01XX08	412903ATB
Lincosamide	lincomycin hydrochloride 0.25g	J01FF02	184201ACH
	lincomycin hydrochloride 0.5g	J01FF02	184203ACH
	clindamycin hydrochloride 0.15g	J01FF01	135401ACH
Macrolide	azithromycin 0.6g(40mg/mL)	J01FA10	112732ASY
	azithromycin 0.9g(40mg/mL)	J01FA10	112733ASY
	azithromycin 0.25g	J01FA10	112701ATB
	erythromycin(enteric coated) 0.25g	J01FA01	154001ACH
	roxithromycin 5g(10mg/mL)	J01GB01	225332ASS
	roxithromycin 5g(50mg/g)	J01FA10	225333AGN
	roxithromycin 15g(50mg/g)	J01GB03	225337AGN
	roxithromycin 0.15g	J01GB11	225301ATB
	roxithromycin 50mg	J01GB07	225302ASS
	roxithromycin 50mg	J01GB07	225302ATB
	doxycycline hydrate 0.1g	J01AA02	149501ACH
	doxycycline hydrate 0.1g	J01AA02	149501ATB
	doxycycline hyclate hydrate 0.1g	J01AA02	149701ACH
	doxycycline hyclate hydrate 0.1g	J01AA02	149701ATB
	oxytetracycline hydrochloride 0.5g	D06AA03	207403ACH
	tetracycline hydrochloride 0.25g	J01AA07	236701ACH
	clarithromycin 2.5g(25mg/mL)	J01FA09	134935ASY
	clarithromycin 3.5g(50mg/mL)	J01FA09	134937ASY
	clarithromycin 0.25g	J01FA09	134901ATB
	clarithromycin 0.5g	J01FA09	134904ATB
	clarithromycin 0.5g	J01FA09	134904ATR
	spiramycin 0.234375g	J01FA02	311000ATB

Quinolone	ciprofloxacin hydrochloride (as ciprofloxacin 0.25g)	J01MA02	134103ATB
	ciprofloxacin hydrochloride (as ciprofloxacin 0.5g)	J01MA02	134105ATB
	ciprofloxacin hydrochloride (as ciprofloxacin 0.5g)	J01MA02	134105ATR
	ciprofloxacin hydrochloride (as ciprofloxacin 1g)	J01MA02	134108ATR
	ciprofloxacin hydrochloride (as ciprofloxacin 0.75g)	J01MA02	134109ATB
	levofloxacin hydrate 0.1g	J01MA12	183201ATB
	levofloxacin hydrate 0.25g	J01MA12	183202ATB
	levofloxacin hydrate 0.5g	J01MA12	183203ATB
	levofloxacin hydrate 0.75g	J01MA12	183205ATB
	ofloxacin 0.1g	J01MA01	203901ATB
	ofloxacin 0.2g	J01MA01	203904ATB
	lomefloxacin hydrochloride 0.1g	J01MA07	184901ATB
	lomefloxacin hydrochloride 0.4g	J01MA07	184903ATB
	lomefloxacin hydrochloride 0.2g	J01MA07	184904ATB
	norfloxacin 0.1g	J01MA06	203301ATB
	norfloxacin 0.2g	J01MA06	203302ACH
	tosufloxacin tosylate 0.15g	J01MA22	242201ATB
	balofloxacin 0.1g	none	428901ATB
	gemifloxacin mesylate (as gemifloxacin 0.32g)	J01MA15	442901ATB
	moxifloxacin hydrochloride (as moxifloxacin 0.4g)	J01MA14	380301ATB
ETC	sulfamethoxazole 0.4g trimethoprim 80mg	J01EE01	311500ATB

Supplementary Table S3. ICD-10 codes for comorbidities including Charlson comorbidities index items and scores

Type of comorbidities	Category	ICD-10 codes	Scores
Included in Charlson comorbidity index	Myocardial infarction	I21, I22, I25.2	1
	Congestive heart failure	I09.9, I11.0, I13.0, I13.2, I25.5, I42.0, I42.5-I42.9, I43, I50, P29.0	1
	Peripheral vascular disease	I70, I71, I73.1, I73.8, I73.9, I77.1, I79.0, I79.2, K55.1, K55.8, K55.9, Z95.8, Z95.9	1
	Cerebrovascular disease	G45, G46, I60-I69, H34.0	1
	Dementia	F00-F03, G30, F05.1, G31.1	1
	Chronic pulmonary disease	I27.8, I27.9, J40-J47, J60-J67, J68.4, J70.1, J70.3	1
	Rheumatologic disease	M05, M06, M31.5, M32-M34, M35.1, M35.3, M36.0	1
	Peptic ulcer	K25-K28	1
	Hemiplegia or paraplegia	G04.1, G11.4, G80.1, G80.2, G81, G82, G83.0, G83.1, G83.2, G83.3, G83.4, G83.9	2
	Diabetes without complication	E10.0, E10.1, E10.6, E10.8	1
	Diabetes with complication	E10.9, E11.0, E11.1, E11.6, E11.8, E11.9, E12.0, E12.1, E12.6, E12.8, E12.9, E13.0 E13.1, E13.6, E13.8, E13.9, E14.0, E14.1, E14.6, E14.8, E14.9	2
	Mild liver disease	B18, K70.0-K70.3, K70.9, K71.3-K71.5, K71.7, K73, K74, K76.0, K76.2-K76.4, K76.8, K76.9, Z94.4	1
	Moderate to severe liver disease	I85.0, I85.9, I86.4, I98.2, K70.4, K71.1, K72.1, K72.9, K76.5-K76.7	3
	Moderate to severe renal disease	I12.0, I13.1, N03.2-N03.7, N05.2-N05.7, N18, N19, N25.0, Z49.0-Z49.2, Z94.0, Z99.2	2
	Any malignancy (including leukemia or lyphoma)	C00-C26, C30-C34, C37-C41, C43, C45-C58, C60-C76 C81-C85, C88, C90-C97	2
	Metastatic solid tumor	C77-C80	6
	Acquired immunodeficiency syndrome	B20-B22, B24	6
Others	Osteoporosis	M80-M82	-
	End stage renal disease	E10.22, E11.22, E12.22, E13.32, E14.22, N18.5, Z99.2 (V001, V003)	-

Supplementary Table S4. HIRA therapeutic codes for transfusion

Type of transfusion	HIRA therapeutic code
Autologous transfusion	X6001-X6008
Allogenous transfusion	X2021, X2022, X2031, X2032, X2091, X2092, X2111, X2131, X2132, X2512, X2515, X9006-9007

Supplementary Table S5. HIRA general name codes for used steroids

Category of steroid	Type of steroid	ATC code	HIRA general name code
Oral steroid	deflazacort 6 mg	H02AB13	140801ATB
	dexamethasone 0.5 mg	H02AB02	141901ATB
	dexamethasone 0.75 mg	H02AB02	141903ATB
	betamethasone 0.25 mg + d-chlorpheniramine 2 mg	H02AB01	296900ATB
	hydrocortisone 10 mg	H02AB09	116401ATB
	hydrocortisone 5 mg	H02AB09	170901ATB
	methylprednisolone 4 mg	H02AB04	193302ATB
	methylprednisolone 1 mg	H02AB04	193305ATB
	prednisolone 5 mg	H02AB06	217001ATB
	triamcinolone 1 mg	H02AB08	243201ATB
	triamcinolone 2 mg	H02AB08	243202ATB
	triamcinolone 4 mg	H02AB08	243203ATB
	fludrocortisone 100 µg	H02AA02	160201ATB
Intravenous steroid	dexamethasone 4 mg	H02AB02	142030BIJ
	dexamethasone 4 mg	H02AB02	142230BIJ
	dexamethasone 5 mg	H02AB02	142232BIJ
	betamethasone 4 mg	H02AB01	116530BIJ
	hydrocortisone 100 mg	H02AB09	171201BIJ
	methylprednisolone 125 mg	H02AB04	193601BIJ
	methylprednisolone 40 mg	H02AB04	193603BIJ
	methylprednisolone 500 mg	H02AB04	193604BIJ
	triamcinolone 10 mg	H02AB08	243336BIJ
	triamcinolone 40 mg	H02AB08	243335BIJ
	triamcinolone 40 mg	H02AB08	243337BIJ

Supplementary Table S6. Risk factors for recurrence when recurrence was defined as an infection requiring over 2-week antibiotics

Variables	Category	Adjusted odds ratio (95% confidence interval)	p-value	Bootstrap adjusted odds ratio (95% confidence interval)	Relative bias (%)
Age	50-59 vs 20-49	2.07 (0.83 - 5.17)	0.117	2.16 (1.04 - 5.74)	5.7
	60-69 vs 20-49	2.43 (1.01 - 5.83)	0.048	2.58 (1.34 - 6.76)	6.8
	70 + vs 20-49	2.29 (0.96 - 5.47)	0.061	2.42 (1.20 - 6.67)	6.9
Comorbidities	Osteoporosis	1.34 (0.93 - 1.93)	0.122	1.15 (0.97 - 1.37)	-52.0
Surgical approach	Cervical anterior vs lumbar posterior	0.86 (0.41 - 1.81)	0.693	0.81 (0.35 - 1.50)	36.3
	Cervical posterior vs lumbar posterior	0.49 (0.06 - 3.67)	0.483	0.01 (0.00 - 0.64)	580.2
	Thoracic anterior vs lumbar posterior	1.50 (0.48 - 4.62)	0.484	1.16 (0.29 - 4.00)	-63.4
	Thoracic posterior vs lumbar posterior	1.85 (1.56 - 2.96)	0.010	1.86 (1.19 - 2.84)	0.9
	Lumbar anterior vs lumbar posterior	1.55 (1.00 - 2.41)	0.051	1.58 (1.15 - 2.13)	3.9
	Multiple vs lumbar posterior	4.16 (2.43 - 7.12)	<0.001	4.45 (2.75 - 6.91)	4.6
Cage		1.67 (1.12 - 2.48)	0.011	1.30 (1.08 - 1.55)	-49.5
Transfusion		2.55 (1.65 - 3.94)	<0.001	1.60 (1.36 - 1.89)	-50.1
Antibiotics for resistant organisms		2.55 (1.80 - 3.63)	<0.001	1.59 (1.37 - 1.84)	-50.2
Systemic steroid	within 2 weeks vs no use	1.26 (0.90 - 1.75)	0.173	1.27 (0.94 - 1.64)	1.8
	Over 2 weeks vs no use	2.30 (1.42 - 3.74)	<0.001	2.30 (1.44 - 3.38)	-0.2

All significant independent variables ($p<0.05$) from the univariable analysis were included in this multivariable model. Relative bias was estimated as the difference between the mean bootstrapped regression coefficient estimates and the mean parameter estimates of multivariable model divided by the mean parameter estimates of multivariable model.

Supplementary Table S7. Risk factors for recurrence when recurrence was defined as an infection requiring over 6-week antibiotics

Variables	Category	Adjusted odds ratio (95% confidence interval)	p-value	Bootstrap adjusted odds ratio (95% confidence interval)	Relative bias (%)
Age	50-59 vs 20-49	2.01 (0.84 - 5.23)	0.129	2.31 (1.14 - 5.43)	20.0
	60-69 vs 20-49	2.53 (1.06 - 6.07)	0.047	2.81 (1.43 - 6.01)	11.2
	70 + vs 20-49	2.47 (1.04 - 5.86)	0.043	2.67 (1.40 - 6.05)	8.7
Surgical approach	Cervical anterior vs lumbar posterior	0.85 (0.41 - 1.80)	0.826	0.80 (0.39 - 1.51)	40.5
	Cervical posterior vs lumbar posterior	0.47 (0.06 - 3.53)	0.499	0.01 (0.00 - 1.00)	508.2
	Thoracic anterior vs lumbar posterior	1.51 (0.49 - 4.67)	0.354	1.26 (0.33 - 1.67)	-44.6
	Thoracic posterior vs lumbar posterior	1.84 (1.15 - 2.94)	0.008	1.84 (1.14 - 2.71)	0.3
	Lumbar anterior vs lumbar posterior	1.55 (1.00 - 2.41)	0.054	1.53 (1.02 - 2.17)	-3.3
	Multiple vs lumbar posterior	4.13 (2.41- 7.07)	<0.001	4.19 (2.67 - 6.77)	1.0
Cage		1.68 (1.13 - 2.49)	0.011	1.30 (1.12 - 1.52)	-49.8
Transfusion		2.52 (1.64 - 3.89)	<0.001	1.61 (1.34 - 1.93)	-48.8
Antibiotics for resistant organisms		2.55 (1.80 - 3.62)	<0.001	1.63 (1.40 - 1.94)	-47.8
Systemic steroid	within 2 weeks vs no use	1.26 (0.91 - 1.75)	0.173	1.27 (0.94 - 1.72)	4.2
	Over 2 weeks vs no use	2.29 (1.41 - 3.71)	<0.001	2.27 (1.55 - 3.53)	-0.9

All significant independent variables (p<0.05) from the univariable analysis were included in this multivariable model. Relative bias was estimated as the difference between the mean bootstrapped regression coefficient estimates and the mean parameter estimates of multivariable model divided by the mean parameter estimates of multivariable model.
